# Emotion Elicitation: A Comparison of Pictures and Films

**DOI:** 10.3389/fpsyg.2016.00180

**Published:** 2016-02-17

**Authors:** Meike K. Uhrig, Nadine Trautmann, Ulf Baumgärtner, Rolf-Detlef Treede, Florian Henrich, Wolfgang Hiller, Susanne Marschall

**Affiliations:** ^1^Media Studies, Film, Television and Audiovisual Media, Tuebingen UniversityTuebingen, Germany; ^2^Department of Clinical Psychology and Psychotherapy, Psychological Institute, University of MainzMainz, Germany; ^3^Chair of Neurophysiology, Centre of Biomedicine and Medical Technology Mannheim, Medical Faculty Mannheim, Heidelberg UniversityMannheim, Germany

**Keywords:** emotion elicitation, film clips, IAPS pictures, media effects, movies

## Abstract

Pictures and film clips are widely used and accepted stimuli to elicit emotions. Based on theoretical arguments it is often assumed that the emotional effects of films exceed those of pictures, but to date this assumption has not been investigated directly. The aim of the present study was to compare pictures and films in terms of their capacity to induce emotions verified by means of explicit measures. Stimuli were (a) single pictures presented for 6 s, (b) a set of three consecutive pictures with emotionally congruent contents presented for 2 s each, (c) short film clips with a duration of 6 s. A total of 144 participants rated their emotion and arousal states following stimulus presentation. Repeated-measures ANOVAs revealed that the film clips and 3-picture version were as effective as the classical 1-picture method to elicit positive emotions, however, modulation toward positive valence was little. Modulation toward negative valence was more effective in general. Film clips were less effective than pictorial stimuli in producing the corresponding emotion states (all *p* < 0.001) and were less arousing (all *p* ≤ 0.02). Possible reasons for these unexpected results are discussed.

## Introduction

There is a longstanding tradition in psychological research of trying to create emotional states in the laboratory for scientific aims. Several methods have been described, including hypnosis (i.e., Bower, 1983) and imagery (e.g., Lang, 1979), music (e.g., Sutherland et al., 1982), facial muscle movements (Ekman et al., 1983), interaction with trained confederates (e.g., Ax, 1953), the Velten-/self-statement technique (repeating phrases with emotional content, Velten, 1968) and even drugs and sleep deprivation (cf. Martin, 1990; Hagemann et al., 1999). Some of these methods bring ethical problems (e.g., drug intake, use of deception) and/or problems of standardization.

One widely used and accepted method for induction of emotional states that does not involve deception and that can be easily standardized, is the use of pictures. The International Affective Picture System (IAPS; Lang et al., 2005) provides such a set of standardized photographs. It has the advantage of being an internationally known method, is available for all researchers to use and normative ratings have been collected and provided by the authors since 1992. IAPS pictures have been used in a wide range of research topics and are typically shown for a duration of 6 s (e.g., Vrana et al., 1988; Bernat et al., 2006; Codispoti et al., 2006; Rhudy et al., 2007). Some studies have also varied the presentation, from subliminal application (Ruys and Stapel, 2008) to 2.5 s duration only (Chiew and Braver, 2011) and up to 8 s (Amrhein et al., 2004). A variation of the usually implemented version, which has shown good results with respect to emotional impact, is the presentation of three successive images with congruent emotional and thematic content for 2 s each (Godinho et al., 2006).

In addition to the IAPS, film clips have also been widely used and accepted as stimuli in the field of emotion research (i.e., McHugo et al., 1982; Gross and Levenson, 1995; Schaefer et al., 2010; Bartolini, 2011). Films have been used for many decades with the first use of film clips for emotion elicitation described in 1930, in a study of the effects of anger, fear and sexual arousal on blood pressure (Scott, 1930). Since then, many studies have used films as emotion elicitors to study phenomena such as sad mood induced smoking behavior (Fucito and Juliano, 2009), emotional modulation of the acoustic startle reflex (Kaviani et al., 1999), the readiness for affective reactions related with frontal brain-asymmetry (Tomarken et al., 1990) and the effect of emotion on eating behavior (Macht et al., 2002).

Films share most of the advantages that pictures offer, like their capability of being standardized and have been argued to be even more advantageous. In contrast to static pictures, films are dynamic and thus thought to be more similar to real life. Film technical elements (Camera-movement, Editing, Sound etc.) can be used to emphasize actions and their emotional meaning (Mikunda, 2002). Thus, Gross and Levenson (1995) state: “Films also have a relatively high degree of ecological validity, in so far as emotions are often evoked by dynamic visual and auditory stimuli that are external to the individual” (p. 88).

Indeed, findings of a previous review attest film clips have the capacity to elicit emotions successfully in most subjects (Martin, 1990). Another review, as well as a meta-analysis, conclude that films effectively induce emotions and even surpass the potential of methods like the Velten technique, hypnosis, or offering presents, especially in relation to the induction of positive emotions (Gerrards-Hesse et al., 1994; Westermann et al., 1996). Surprisingly, none of these comparisons included static pictures as emotional stimuli. The only studies to our knowledge that have compared pictures and films, aimed to provoke the very specific emotional reactions to sexual stimulation and found films to be more effective (Heiman, 1980; Julien and Over, 1988). However, even though there are many theoretical arguments in favor of films, very little empirical evidence can be found.

To address this lack of research we developed a set of film clips that is formally comparable to the already established IAPS pictures, by reducing each film clip to 6 s in duration. We exploited the advantages of films in accordance with findings from the field of film studies, and eliminated potential weaknesses of current databases regarding within set comparability to ensure reliability (e.g., the varying lengths of film clips).

Emotional effects induced by presentation of the stimuli were quantified using a paper-and-pencil version of the Self-Assessment Manikin scale (SAM; Lang et al., 2005). Although implicit measures of affective processing prove to be a highly valuable means to get a profound insight into non-conscious affective processing, in this study we concentrated on conscious effects detectable by using explicit ratings only.

The aim of this study was to test the effectiveness of this film set regarding modulatory effects on emotion by comparing it to that of the established IAPS dataset. We hypothesized that short film clips would be at least as effective as static pictures in inducing positive and negative emotional states. Secondly, we aimed to test the effectiveness of a third condition consisting of three emotionally congruent pictures shown successively, as this may be a promising variant to the classic picture paradigm for successful induction of emotions.

## Materials and methods

### Participants

A total of 144 students participated voluntarily in the experiment, who were recruited through locally placed notices. Five subjects had to be excluded from the analysis due to either incorrectly completing the rating form or extreme mood states prior to the experiment, leading to biased ratings. Out of the remaining 139 participants, with a mean age of 25 (SD 6) years, 59.7% were female and 40.3% were male. The overall pre study mood was slightly positive (Mean 6.3, SD 1.3, range 3–9), and participants were relatively calm, but also wide-awake and attentive (Mean 4.4, SD 1.3, range 1–7). This study was performed according to the Declaration of Helsinki and was approved by the local ethics committee.

### Stimulus material

For the film condition, we selected fictional live-action films, only excluding those that were expected to cause side effects (i.e., that were produced in the period of silent film, shot in black-and-white or that used outdated technologies to create film-effects). Furthermore, only mainstream films were chosen which followed the classic Hollywood film style, the so called continuity system (Bordwell, 2006), as they conventionally aim for viewers to experience quite specific and intense emotional responses. They are also produced according to a standardized film style that promotes their emotional impact on every filmic level—using specific lighting, camera-angles or sound, standardized scenes and genre-specific actors. The selected films therefore allow for a database that fulfills the requirement of comparability of the film clips *within* the database.

Before choosing the films we conducted a preliminary study with 27 participants who were asked to recollect films or film scenes that they remembered to be particularly moving. These films, along with mainstream films from three local video stores, were analyzed for their emotional content from a scientific filmologic point of view. This was based on the analysis, whether the clips contained coherent emotional content (either positive or negative) in its subdivisions (aesthetic/technical aspects like light or music, action and characters, symbolism, and production aspects). Finally, 80 film clips were selected, all of them produced in Hollywood Style between the time span of 1979 and 2007. The film clips were classified for emotional effect^1^ and underwent preliminary testing with six participants. Forty of the film clips were categorized as stimuli that induce positive emotions (amusement, happiness, love), and 40 as stimuli that induce negative emotions (fear, disgust, sadness, see Table 1).

**Table 1:** **Description of film clips**.

**No**.	**Emotion (Valence)**	**Source film**	**Clip description**
1	Disgust (negative = N)	*Saving Private Ryan* (Steven Spielberg 1998)	Medium shot of a soldier lying on the floor, bleeding heavily from an abdominal wound. Other soldiers are kneeling beside him trying to make the bleeding stop.
2	Fear (N)	*Scream* (Wes Craven 1996)	A young girl is running over a lawn fleeing from a masked man wielding a knife. The man catches up. Sharp orchestral music drowns her fearful moaning
3	Fear (N)	*Monster* (Patty Jenkins 2003)	Extreme close-up of bound hands, then a shot of a woman lying face down in a car. A man is kicking her, cursing and demanding that she screams. The woman screams and moans, contorted in pain.
4	Disgust (N)	*Joan of Arc* (Christian Duguay 1999)	Extreme close-up of an arrow sticking into an abdominal wound. Then a close-up of the pain contorted face of a female knight, followed by another close-up of the bleeding wound. Someone is trying to pull the arrow out of the wound. In the background, loud moaning of the injured woman can be heard.
5	Sadness (N)	*The English Patient* (Anthony Minghella 1996)	A man is leaning over a crying woman in a tent to protect her. You can hear explosions and people screaming. The woman is desperately crying: “He is dead.” A cut shows people running around in panic, bombs are falling down around them.
6	Amusement (positive = P)	*What Women Want* (Nancy Meyers 2000)	Medium shot of a man. He is trying to get his legs into a woman's stockings. He has an anti-pimple plaster on his nose.
7	Amusement (P)	*Bruce Almighty* (Tom Shadyac 2003)	Medium shot of a man who is singing and walking through the streets in a clownish fashion to the rhythm of the 90's song "I've got the Power."
8	Happiness (P)	*Sister Act* (Emile Ardolino 1992)	Nuns in a choir are singing and clapping to a rhythmic song. A sister is singing the solo part bashfully and is visibly happy about it afterwards.
9	Love (P)	*James Bond 007—Die another day* (Lee Tamahori 2002)	Seemingly through the lens of a telescope, we see a bond girl coming out of the water in slow motion, wearing a bikini. Apart from the sound of her plunging through water, sensual violin music can be heard.
10	Happiness (P)	*Big Fish* (Tim Burton 2003)	A window is opened and reveals a view of a man in a suit who is standing in the middle of a field of flowers. A cut to the window shows his sweetheart, she is smiling, touched.
11	Sadness (N)	*Coach Carter* (Thomas Carter 2005)	Close-up of a basketball-player's sad face. Another close-up shows his disappointed team-mates. Blower's music accompanies the scene.
12	Fear (N)	*Kill Bill II* (Quentin Tarantino 2004)	A woman is lying in a dark wooden box, only a flashlight is lighting her desperate face. Only her breath can be heard and the noise of the earth that is falling on the box in which she is trapped.
13	Disgust (N)	*Blade* (Stephen Norrington 1998)	In a crowded room that is completely smothered in red paint, a darkly dressed man shoots at a vampire, who then crumbles with a gurgling noise.
14	Sadness (N)	*Braveheart* (Mel Gibson 1995)	A close-up shows the sad face of a man. A following close-up shows a hatchet falling down in slow motion. After that a close-up of the man's hand is seen, slowly opening whilst he is dying. Supporting violin music accompanies the scene.
15	Sadness (N)	*Gladiator* (Ridley Scott 2000)	Close-up of a man crying desperately, kneeling on the floor and reaching up with his hand pleadingly.
16	Amusement (P)	*Meet the Parents* (Jay Roach 2000)	A man in pajamas enters his bathroom and finds a cat sitting on the toilet. He apologizes then leaves the room looking confused.
17	Love (P)	*Bridget Jones's Diary* (Sharon Maguire 2001)	Close-up of the face of a woman, who is nodding, touched. A close-up shows her walking toward a man and falling into his arms. Falling snowflakes and harmonious piano music underlie the romantic scene.
18	Happiness (P)	*Dances with Wolves* (Kevin Costner 1990)	Cut from the picture of a sunset to a campfire. There's a man sitting and eating in an otherwise deserted landscape.
19	Amusement (P)	*Mr. Bean's Holiday* (Steve Bendelack 2007)	A man is decorating a British bobby with flowers. In the background people can be heard laughing.
20	Love (P)	*American Pie* (Paul Weitz 1999)	A young couple are lying naked under a blanket by the lake. Romantic pop music accompanies the scene.
21	Happiness (P)	*Amélie* (Jean-Pierre Jeunet 2001)	The recording of a blurred hand-held camera shows a young couple in fast motion, driving through the streets of Paris on a scooter.
22	Love (P)	*Erin Brockowich* (Steven Soderbergh 2007)	A baby is seen sleeping peacefully in his bed and someone places covers over him.
23	Amusement (P)	*Bruce Almighty* (Tom Shadyac 2003)	A man looks into his bathroom and sees his dog use the toilet then flush it afterwards. A look of self-satisfaction crosses his face. Happy music accompanies the scene.
24	Love (P)	*When Harry Met Sally* (Rob Reiner 1989)	Close-up of a man leaning over to his wife and kissing her. In the background you can hear the song "Auld Lang Syne," which traditionally accompanies the change of the year.
25	Amusement (P)	*Pirates of the Caribbean* (Gore Verbinski 2003)	A pirate is standing on the mast pole of his ship staring toward the port. Only through the backward movement of the camera we can see that the ship has already sunk and only the mast pole is sticking out of the water.
26	Fear (N)	*Big Fish* (Tim Burton 2003)	A boy is walking toward the entrance door of a dark house. Dark shadows and tense music accompany his step. Through a rapid camera movement the filming switches to a close-up of a scary witch with an eye-patch, as she rapidly opens the door.
27	Disgust (N)	*The Hills have Eyes* (Alexandre Aja 2006)	A man lights a match and finds himself in a container full of mutilated corpses. He screams and tries to free himself in panic. The filming switches several times between the man and his subjective point of view.
28	Sadness (N)	*Finding Neverland* (Marc Forster 2004)	A small boy sits on a park bench next to a man dressed in black. The sad face of the boy is shown close-up. He holds his head down and swallows. Then he looks up and looks at the man helplessly, with tears in his eyes.
29	Fear (N)	*Saving Private Ryan* (Steven Spielberg 1998)	Soldiers are seen throwing themselves behind a sand-bunker and protectively holding their heads down, whilst bullets hit the ground next to them. A soldier cries.
30	Fear (N)	*The Texas Chain Saw Massacre* (Marcus Nispel 2003)	Close-up of a man lying on the floor and saying desperately: “I am your friend!,” then another man strikes his head with a hammer.
31	Happiness (P)	*Coach Carter* (Thomas Carter 2005)	A basketball flies into a basket in slow motion. The audience jump up and down in their seats jubilating. Dramatic music accompanies the scene.
32	Love (P)	*Moulin Rouge* (Baz Luhrmann 2001)	A man and a woman covered in a bed sheet hug each other in front of an open window. Two doves are sitting on the ledge. A cut shows the woman lying in bed listening to the man reading to her from a book. A love song sung by both of them accompanies the scene.
33	Amusement (P)	*Finding Neverland* (Marc Forster 2004)	A man in a suit and bow tie sits at a dinner table in fine company and shows the children who are present a magic-trick.
34	Love (P)	*Along came Polly* (John Hamburg 2004)	A medium close-up shows a young woman dancing erotically with a man, to salsa music.
35	Happiness (P)	*The Family Stone* (Thomas Bezucha 2005)	A close-up shows a young woman dancing lively and singing to cheerful music.
36	Disgust (N)	*Die Hard* (John McTiernan 1988)	A man is lying on the floor and tries to drag himself into a room, moaning. A close-up shows his wounded leg leaving a trail of blood whilst he tries to save himself.
37	Sadness (N)	*Saving Private Ryan* (Steven Spielberg 1998)	A man is standing in a military cemetery between graves. Leading orchestral music accompanies the scene.
38	Sadness (N)	*About Schmidt* (Alexander Paine 2002)	A close-up shows an old man crying. Leading orchestral music accompanies the scene.
39	Disgust (N)	*Seven* (David Fincher 1995)	An injured, extremely emaciated man with wide open eyes is lying in a scruffy bed and frantically fidgeting to keep himself alive.
40	Disgust (N)	*Naked Lunch* (David Cronenberg 1991)	A close-up shows a giant dead insect. It leaves a pool of dried up blood and its organs are pouring out, as a cursing man stuffs it into a bag.
41	Disgust (N)	*Alien* Ridley Scott 1979)	A close-up shows the frightened face of a man, followed by a close-up of an alien filmed from below. Another cut shows the alien attacking the panic-stricken, screaming man.
42	Sadness (N)	*Gladiator* (Ridley Scott 2000)	A close-up from a bird's perspective shows a dying gladiator lying on the ground. A following cut shows the close-up of a grieving woman looking down on him. A musical dirge accompanies the scene.
43	Disgust (N)	*The Hills have Eyes* (Alexandre Aja 2006)	A blood-smeared man pulls a rod out of the head of a mutilated corpse and rams it through the neck of a loud whining zombie.
44	Sadness (N)	*Moulin Rouge* (Baz Luhrmann 2001)	A top shot shows a loudly weeping young man who kneels on a bed of flowers and holds his dead lover in his arms. Slow orchestral music accompanies the scene.
45	Fear (N)	*Saw* (James Wan 2006)	A close-up shows a mother and her daughter sitting bound and gagged in front of a bed. A hooded, black-clad figure comes into the picture and is seen approaching the child with a stethoscope. The child cries and the mother pleads with the figure to leave her child alone.
46	Happiness (P)	*The Miracle of Bern* (Sönke Wortmann 2003)	A football player shoots the ball toward the goal and scores. The audience jump to their feet cheering. In the background the commentator cheers. Increasing dramatic music accompanies the scene.
47	Happiness (P)	*Big Fish* (Tim Burton 2003)	A long shot shows a man driving a red sports car on a highway. Upbeat country music accompanies the scene.
48	Amusement (P)	*What Women Want* (Nancy Meyers 2000)	A young woman in an evening dress observes herself in the mirror and mentally comments: "I can't believe I'm wearing this on my last night as a virgin—or better said—taking it off." Whereupon her father comes into the picture and is seen falling backwards out of his chair.
49	Happiness (P)	*Harry Potter* (Chris Columbus 2001)	A young boy is standing in the courtyard of a snow-covered school and releases a white owl into the air. The camera follows the owl's flight, which is accompanied by solemn orchestral music.
50	Love (P)	*There's Something About Marry* (Bobby Farrelly, Peter Farrelly 1998)	A close-up shows a smiling woman, followed by a close-up of a smiling man walking toward her. His gait is accompanied by a rising musical tone. They kiss and the sound explodes into joyous music.
51	Sadness (N)	*E.T*. (Steven Spielberg 1982)	A close-up shows a little boy with tears in his eyes, saying goodbye to ET. Dramatic orchestral music accompanies the scene.
52	Sadness (N)	*William Shakespeare's Romeo and Juliet* (Baz Luhrmann 1996)	A close-up shows a crying wounded man holding his dying friend in his arms.
53	Fear (N)	*The Sixth Sense* (M. Night Shyamalan 1999)	A little boy is sitting anxious and gasping in a tent. The camera pans up and shows the tent slowly opening from the outside. Dramatic orchestral music begins.
54	Fear (N)	*Silence of the Lambs* (Jonathan Demme 1991)	A top shot shows an anxious woman praying. "I want my mommy." Followed by a cut to a man who looks down on her smiling and amused.
55	Disgust (N)	*Seven* (David Fincher 1995)	The camera moves slowly toward the back of a very corpulent man sitting at a table. A medium close-up of his profile shows that he is dead, with his face in his plate.
56	Love (P)	*Mission: Impossible* (Brian De Palma 1996)	A close-up shows a young couple looking at each other with love and then kissing. The following close-up shows the couple kissing in the middle of a park, by a river, with children playing in the background. Harmonious music accompanies the kiss.
57	Amusement (P)	*When Harry Met Sally* (Rob Reiner 1989)	Close-up of a woman pretending to have an orgasm at a restaurant. Reverse shot to her partner who looks around embarrassed and smiles at the other guests.
58	Love (P)	*Gangs of New York* (Martin Scorsese 2002)	An extreme close-up shows a woman's hand, which is carefully placed onto a man's bare chest. The quiet breathing of the man is heard.
59	Happiness (P)	*Sense and Sensibility* (Ang lee 1995)	The camera pans over a green landscape and into a detached property. Birdsong is heard. The calm singing of a woman accompanies the scene.
60	Amusement (P)	*Sister Act* (Emile Ardolino 1992)	A stout nun is seen, playfully dancing in a disco to peppy Rock N Roll music.
61	Happiness (P)	*American Pie* (Paul Weitz 1999)	Young people are playfully singing a swing music song in a choir.
62	Amusement (P)	*Mr. Bean's Holiday* (Steve Bendelack 2007)	A man standing beside his bed puts his teddy bear to sleep in a shoebox. People are heard laughing in the background
63	Amusement (P)	*Bruce Almighty* (Tom Shadyac 2003)	A man stands on the railing of his balcony and draws the moon closer with an invisible rope. A song by Tom Jones accompanies the scene.
64	Happiness (P)	*Fried Green Tomatoes* (Jon Avnet 1991)	A long shot shows a classic car driving slowly along a deserted piece of land. Contemplative orchestral music accompanies the scene.
65	Love (P)	*Gangs of New York* (Martin Scorsese 2002)	A close-up shows a couple kissing passionately. The camera slowly moves away. Their breathing can be heard. Slow bagpipe music accompanies the scene.
66	Sadness (N)	*Angela's Ashes* (Alan Parker 1999)	A man puts a dead baby into a cradle. A close-up shows the sad face of a little boy followed by a close-up of a crying woman.
67	Disgust (N)	*The Hills have Eyes* (Alexandre Aja 2006)	A close-up shows a mutilated corpse with an American flag on a stick stuck into it. Followed by a cut to a choking man. Followed by another close-up of the corpse.
68	Fear (N)	*American History X* (Tony Kaye 1998)	A close-up shows a man lying on the floor biting into a curbstone. A wide shot and a following medium close-up show another man taking off and stepping on the head of the man lying on the ground. A loud "snapping" sound is heard.
69	Fear (N)	*Panic Room* (David Fincher 2002)	A woman and her daughter are fleeing from a man. They are seen fleeing from room to room inside a house. Fast cuts, changing camera angles and settings accompany the flight. Dramatic orchestral music accompanies the scene.
70	Disgust (N)	*Silence of the Lambs* (Jonathan Demme 1991)	An extreme close-up shows the open mouth of a man who has a larva removed with tweezers. Dramatic violin music accompanies the scene.
71	Happiness (P)	*Mission: Impossible* (Brian De Palma 1996)	A landscape shot shows a sunset behind a mountain backdrop. The image fades into a shot of a car driving along a mountain road. Rhythmic swing music accompanies the scene.
72	Love (P)	*Pirates of the Caribbean* (Gore Verbinski 2003)	A close-up shows a young couple standing by the sea looking at each other with love. The man kisses his beloved. The camera pulls backwards, leaving them behind. Solemn orchestral music accompanies the scene.
73	Amusement (P)	*Along came Polly* (John Hamburg 2004)	A man is seen playfully dancing a silly dance to salsa music, in front of a crowd.
74	Love (P)	*William Shakespeare's Romeo and Juliet* (Baz Luhrmann 1996)	A young man and a young woman are smiling shyly at each other through the glass of an aquarium. A slow love song accompanies the scene.
75	Amusement (P)	*Fried Green Tomatoes* (Jon Avnet 1991)	A woman courageously smears a serious-looking man's face with a spoonful of chocolate cream. She and another woman—who are completely smothered in cream—hysterically laugh about it.
76	Fear (N)	*Saving Private Ryan* (Steven Spielberg 1998)	Screaming soldiers are running around wildly and trying to hide as shots are fired all around them.
77	Sadness (N)	*The Family Stone* (Thomas Bezucha 2005)	A woman raises her arms in a helpless gesture. A young crying man approaches and hugs her.
78	Disgust (N)	*Kill Bill II* (Quentin Tarantino 2004)	An extreme close-up shows the resolute-looking eyes of a blood smeared woman. Slow, rhythmic orchestral music begins the scene. This is followed by a close-up cut showing the woman quickly pulling out another woman's eye. The wounded woman cries out loud.
79	Sadness (N)	*Philadelphia* (Jonathan Demme 1993)	A close-up shows a man who is fighting back tears. Slow, supporting, popular music accompanies the scene.
80	Fear (N)	*Monster* (Patty Jenkins 2003)	A medium close-up shows a man holding his hands above his head. He is threatened by a woman with a gun, which prompts him to kneel down. He begs her not to kill him.

For the 1-picture condition (1pic), 40 images were selected that induce positive emotions and 40 images that induce negative emotions. Fifty five of these were gathered from the IAPS database^2^. Additional pictures with more human body related contents, independent of the IAPS pictures, were needed for the purpose of a following study based on the data obtained here. Therefore, 11 additional pictures were selected by the authors for that study and another 14 pictures were included that have already been used in a previous study (Godinho et al., 2006). These pictures showed positive contents (amusement and love) comparable to that shown in IAPS pictures.

For the 3-picture condition (3pic), 40 image sequences for the induction of a positive emotion state and 40 image sequences for the induction of a negative emotion state were produced, each image sequence consisting of three emotionally congruent images. As every stimulus in this condition consisted of three single images, there were many more images needed than were available. For this reason, every image in this condition was used twice (mirror-inverted once), so that 120 pictures had to be selected—60 pictures for the induction of a negative emotion state and 60 pictures for the induction of a positive emotion state. Among these, 90 pictures were chosen from the IAPS^3^, 26 pictures from the Godinho et al. (2006) study and four pictures were selected additionally by the authors.

### Assessment of emotional modulation

A paper-and-pencil version of the Self-Assessment Manikin scale (SAM; Lang et al., 2005) was used to acquire (dis)pleasure and arousal ratings of the subjects after presentation of the pictures and films. Both are ordinal scales for valence and arousal, illustrated by little manikins depicting five steps from unhappy to happy (valence) and from calm to excited (arousal), respectively. Subjects indicated how they felt by marking the corresponding symbol or the place between two symbols, resulting in a 9-point-rating scale with “5” representing neutral valence and average arousal. Each stimulus was followed by the rating instruction, resulting in a rating for each film, picture and picture sequence.

### Procedure and experimental design

Information about the nature as well as the time and place of the experiment was provided on locally posted notices as well as the Internet. A total of four experimental sessions with 35 subjects on average took place in the lecture room of the Institute of Film Studies, in the University of Mainz, Germany. After being welcomed by the experimenters, subjects were informed about the experimental procedure and were provided with the SAM rating forms. Participants were instructed in particular to look intensely at the presented stimuli, not to look away even if they might want to and to let the pictures and films affect them. Immediately prior to the experiment, participants were asked to rate their momentary mood and arousal via the 9-point SAM rating scale. After the room was darkened, the films and pictures were shown on a big screen. There was a short break after the first half of the experiment. At the end of the presentation, all of the SAM scales were collected by the experimenters. Altogether, the experiment lasted approximately 1 h.

There were three experimental conditions: one image shown for 6 s (1pic), three consecutive images of the same valence and with similar content presented for 2 s each (3pic) and a short film clip of 6 s (film). The presentation time was therefore the same for all three conditions. Altogether, 240 stimuli (80 in each condition) were to be rated. As the authors expected this amount to be too demanding for a single experimental session (in the sense that subjects' interest and concentration would decrease), the picture and film stimuli were split into two parallel sets of 120, with 40 stimuli per condition (i.e., 20 negative film clips, 20 positive film clips, 20 negative single pictures, 20 positive single pictures, 20 negative picture sequences and 20 positive picture sequences). Seventy three subjects were presented set 1 and 71 were presented set 2. Within each set, the stimuli were grouped into blocks of 20 pictures or picture sequences or film clips each. Every five consecutive stimuli within each block were of the same emotional valence (positive or negative). The conditions (i.e., stimulus types) were arranged in different orders within the two sets to avoid order effects.

### Data analysis

The valence and arousal ratings (dependent variables) were averaged by stimulus quality (positive, negative). To examine the effects of the *condition* (1pic, 3pic, film), repeated-measures analyses of variance (ANOVA) with *condition* as within-subject factor, were conducted. Although the authors aimed to distribute all stimuli equally in the two sets, effects of the set could not be ruled out a priori. Therefore, *set* was initially included as a predictor (between-subject factor) for both variables; yet the main effects of the *set* on valence ratings were not significant (*p* = 0.197). The condition*set interaction effect on the valence ratings was significant for negative stimuli (*p* ≥ 0.029, partial η^2^ = 0.03), but follow-up comparisons were not significant for either pair (all *p* ≥ 0.233). For positive stimuli the interaction was only significant by trend and the effect size was small (*p* = 0.05, partial η^2^ = 0.02). We consequently removed *set* as a predictor for the valence ratings. However, for the arousal ratings, the condition*set interaction effects as well as follow-up comparisons were significant, therefore separate repeated-measures ANOVAS were calculated for set 1 and 2.

*Gender* was included in the final model as a between-subject factor for both variables, as effects of gender on valence and arousal ratings are well-known (cf. norm data by Lang et al., 2005).

To overcome potential sphericity problems, Greenhouse-Geisser-corrected values are reported. If not otherwise reported, in the case of significant results, paired *t*-tests were calculated with Bonferroni adjustment for multiple comparisons.

Partial eta-squared (partial η^2^) was used as the effect size for *F*-tests and Cohen's *d* was used for mean comparisons. Effect sizes are interpreted following Cohen's guidelines (1977): a partial η^2^ of 0.01, 0.06, and 0.14 as well as a *d* of 0.2, 0.5, and 0.8 are considered as small, medium and large, respectively.

## Results

The statistics of all results described below is shown in our Supplementary Material.

### Valence ratings

#### Positive and negative stimuli—changes compared to baseline

Positive stimuli, regardless of whether picture or film, resulted in positive SAM valence ratings. Yet, with a mean valence rating of 6.4 following the positive pictures and films (SD ≤ 0.8 for all three conditions), there was no significant change found in comparison to the mean baseline mood state (6.3 ± 1.3 SD). This contrasts with the valence ratings following negative stimuli, which were clearly below and significantly different from the baseline measures (3pic: 2.9 ± 0.9; 1pic: 3.0 ± 0.9; film: 3.8 ± 0.9).

#### Positive stimuli—effects of stimulus condition and gender

**Figure 1:**
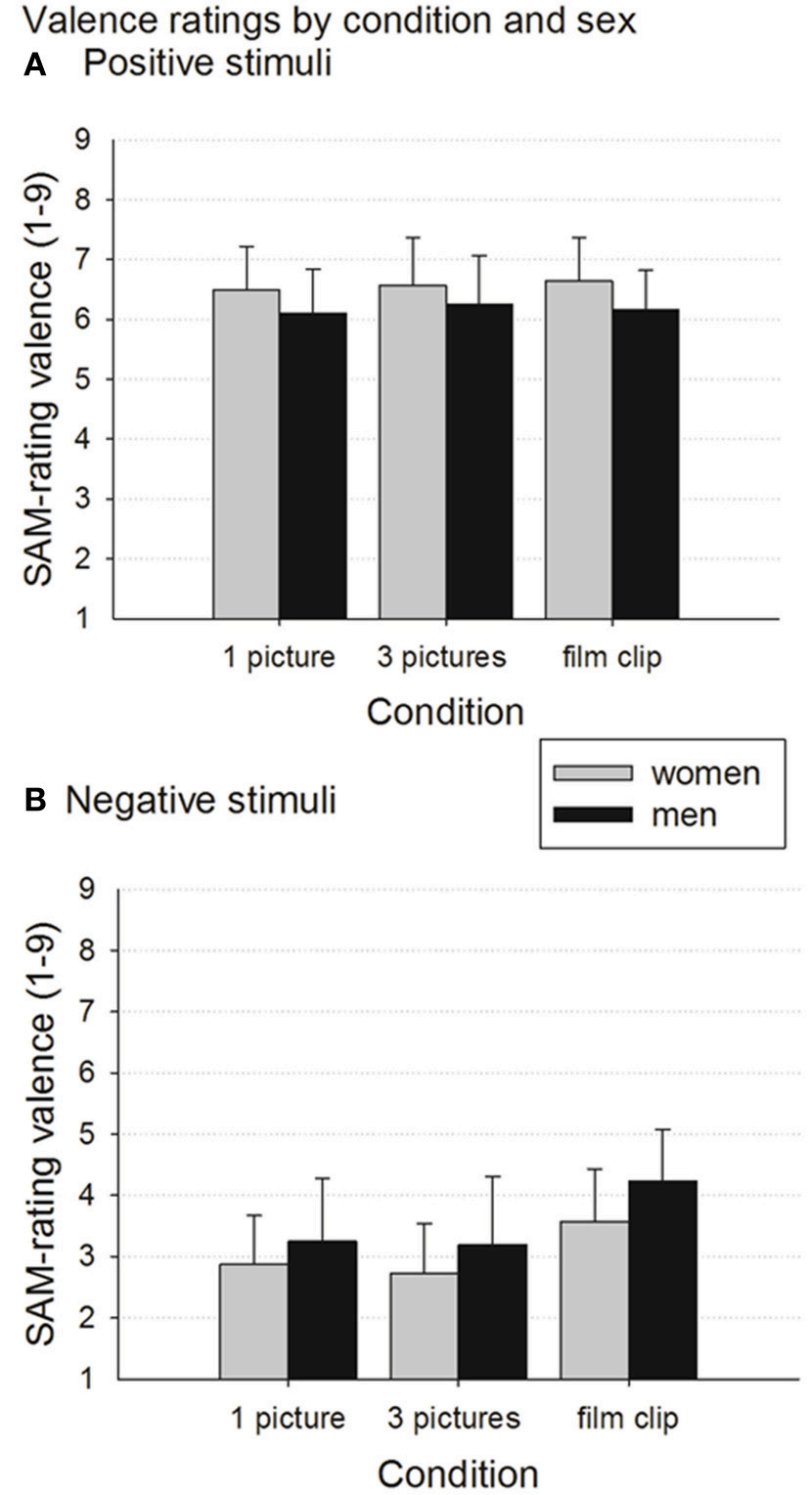
**Valence ratings by *stimulus condition* and *gender***. Data presented as group means ± standard deviations. Higher ratings represent more positive emotion states. For results of significance tests see text. **(A)** Positive stimuli. **(B)** Negative stimuli.

The repeated-measures ANOVA for positive stimuli (see Figure 1A) yielded a trend for a main effect of the *stimulus condition*, *F*_(1.8, 248.9)_ = 2.683, *p* = 0.076; partial η^2^ = 0.019. Follow-up comparisons yielded a slight difference between the 1pic condition and the 3pic condition, which just failed to reach significance (*p* = 0.051, *d* = 0.13), with the 1pic condition evoking slightly lower valence ratings. A further finding was that the main effect of *gender* was significant, *F*_(1,137)_ = 12.124, *p* = 0.001, partial η^2^ = 0.081, with women rating their emotions following the presentation of stimuli with positive contents significantly more positively than men.

#### Negative stimuli—effects of stimulus condition and gender

The ANOVA for negative stimuli suggested the *stimulus condition* main effect was highly significant, *F*_(1.6,223.5)_ = 196.3, *p* < 0.000, partial η^2^ = 0.589. Emotion ratings following negative stimuli were significantly more negative in the 3-picture condition than in the film condition (*p* < 0.000, *d* = 0.97) and the 1-picture condition (*p* = 0.030, *d* = 0.11). In addition, the 1-picture condition yielded more negative emotion ratings than the film condition (*p* < 0.000, *d* = 0.89). The main effect of *gender* was also significant, *F*_(1,137)_ = 12.009, *p* = 0.001, partial η^2^ = 0.081, with women generally reporting stronger negative emotion states than men, following negative stimuli. The stimulus condition*gender interaction was also significant, *F*_(1.631, 223.462)_ = 4.272, *p* = 0.022, partial η^2^ = 0.03, thus moderating the aforementioned relationships. The difference in valence ratings between the film condition and the 1pic and 3pic condition, respectively, was highly significant for both men and women with large effect sizes (all *p* < 0.000, all *d* ≥ 0.83), but the difference between the 1-picture and the 3-picture condition was significant for women only, with a small effect size (*p* < 0.000, *d* = 0.18) (see Figure 1B).

### Arousal ratings

#### Positive stimuli—set 1

**Figure 2:**
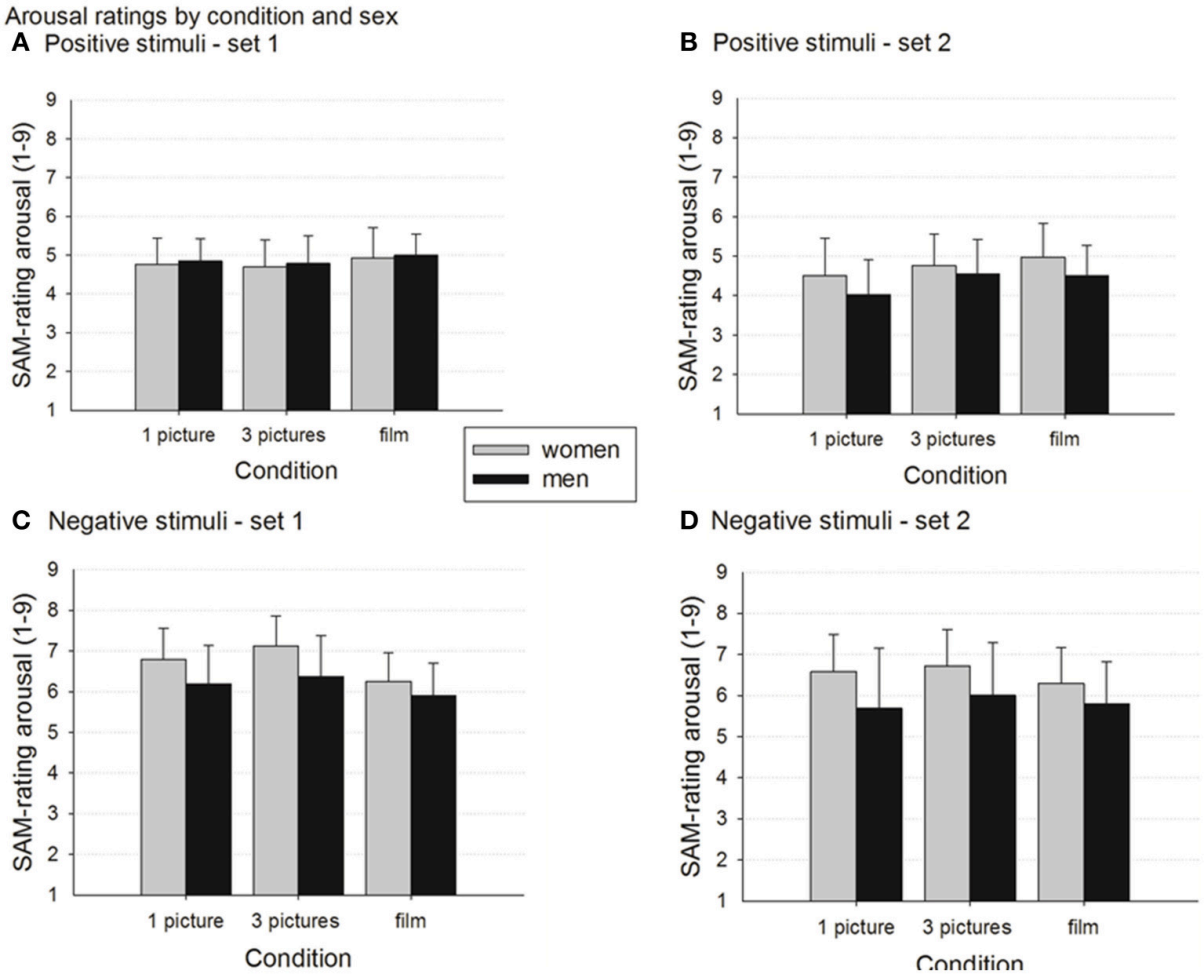
**Arousal ratings by *stimulus condition* and *gender***. Data presented as group means ± standard deviations. Higher ratings represent higher arousal. For results of significance tests see text. **(A)** Set 1 positive stimuli. **(B)** Set 2 positive stimuli. **(C)** Set 1 negative stimuli. **(D)** Set 2 negative stimuli.

In set 1, the ANOVA yielded a significant main effect of the *stimulus condition*, *F*_(1.6,110.2)_ = 5.9, *p* = 0.007, partial η^2^ = 0.079 (see Figure 2A). Follow-up comparisons show that the film condition yielded higher arousal ratings than the 3pic condition (*p* = 0.021, *d* = 0.33). No further significant effects were found.

#### Positive stimuli—set 2

A significant main effect of the *stimulus condition*, *F*_(1.8,122.8)_ = 21.8, *p* < 0.000, partial η^2^ = 0.246 was also found in set 2 with films (*p* < 0.000, *d* = 0.53) and the 3pic condition (*p* < 0.000, *d* = 0.41), eliciting significantly higher arousal ratings than the 1pic condition, respectively. In addition, ANOVA yielded a trend for a *gender* effect, *F*_(1,67)_ = 3.9, *p* = 0.052, partial η^2^ = 0.055. Women's ratings were slightly higher than men's (see Figure 2B).

#### Negative stimuli—set 1

The ANOVA with set 1 data yielded a significant main effect of the *stimulus condition*, *F*_(1.6,111.1)_ = 58.0, *p* < 0.000, partial η^2^ = 0.460 with all pairwise comparisons being significant at level *p* < 0.000 (1pic-3pic *d* = 0.3, 1pic-film *d* = 0.52, 3pic-film *d* = 0.82). The main effect of *gender* was also significant, *F*_(1,68)_ = 9.4, *p* = 0.003, partial η^2^ = 0.121, with women being more aroused by the negative stimuli than men.

A significant stimulus condition*gender interaction, *F*_(1.6, 111.1)_ = 5.3, *p* < 0.010, partial η^2^ = 0.072 complemented the main effects. For both men and women, the 3pic condition yielded the highest arousal ratings, followed by the 1pic condition which was then followed by the film condition. Yet, these differences are somewhat stronger in women than in men (3pic-film *p* < 0.000, *d* = 1.22 [women] vs. *p* = 0.001, *d* = 0.52 [men]; 3pic-1pic *p* < 0.000, *d* = 0.45 [women] vs. *p* = 0.012, *d* = 0.19 [men]; 1pic-film *p* < 0.000, *d* = 0.73 [women] vs. *p* = 0.007, *d* = 0.33 [men]; see Figure 2C).

#### Negative stimuli—set 2

The ANOVA with set 2 data also yielded a significant main effect of the *stimulus condition, F*_(1.4, 96.1)_ = 5.2, *p* = 0.015, partial η^2^ = 0.071. Follow up comparisons revealed that in set 2, as in set 1, the 3pic condition led to highest arousal ratings, significantly higher than the ratings in the 1pic condition (*p* = 0.002, *d* = 0.17) and in the film condition (*p* = 0.020, *d* = 0.33), respectively (see Figure 2D). Also, the main effect of *gender* was significant, *F*_(1,67)_ = 8.9, *p* = 0.004, partial η^2^ = 0.118. Women showed higher arousal than men following the presentation of the set 2 negative stimuli.

## Discussion

This study aimed to compare pictures and film clips with respect to their capacity to elicit emotions, as currently this has not been systematically investigated. We also aimed to assess the effectiveness of a set of short film clips that were developed for this purpose, as well as the effectiveness of a 3-picture condition. The short film clips produced the corresponding emotion: By means of absolute ratings, negative film clips led to negative emotion ratings and positive film clips led to positive emotion ratings. However, in relation to baseline valence ratings, only modulation following presentation of negative content was significant. Overall they were similar to pictures at evoking positive emotion and arousal states but pictures were even more efficient in evoking negative emotion. The following sections describe and discuss the results in more detail.

It is important to note in advance, that the meaning of the term “emotion” as used in this report is restricted to the conscious perception of an affective state and its modulation induced by the visual (pictures) and multimodal (film clips) stimuli, expressed as explicit measure that was captured by SAM ratings of valence and arousal. The authors are aware of the fact that there are several definitions of “emotions” in a much broader setting, and concepts of how emotions may be separated from, or linked to consciousness (Rolls, 1992), how emotions may emerge from subcortical “raw affect” through limbic and neocortical processing (Solms and Panksepp, 2012; Walla and Panksepp, 2013), which is underlined by very recent evidence that affective processing is indeed taking place at subcortical level already (Schepman et al., 2015). Ongoing investigations of the role of autonomic responses, cellular and network level responses in experimental models of emotion substantially improved our understanding on how emotion processing might occur (LeDoux, 1992).

### Effects of gender

It is well-known that women are emotionally more expressive (Kring and Gordon, 1998), at least with respect to negative cues (Bradley et al., 2001b). Therefore, it is not surprising that some studies have found more pronounced emotion ratings for women (e.g., Gross and Levenson, 1995; Hagemann et al., 1999), but not all studies found this (e.g., Philippot, 1993). Our data clearly suggests an effect of gender in the sense that women's subjective feelings (valence) are modulated stronger by affective stimuli.

### Emotion elicitation (valence and arousal ratings)

With regard to the positive range of emotionality, short film clips and the 3-picture variant were as effective in creating the desired emotional valence as the classical 1-picture condition. For arousal ratings the two stimulus sets had to be analyzed separately, thus common conclusions among the sets must be drawn cautiously. Our results suggest that the pictures with positive content evoke somewhat lower arousal than the film clips, although this effect was not consistently significant.

Looking at the negative stimuli, our results suggest that the film clips were clearly less effective than both picture variants in producing negative emotions. This corresponds to the finding, that negative film clips led to lower emotional arousal than at least the negative 3pic variant (this difference was statistically significant in both sets whereas the difference between film clips and the 1pic variant was significant in set 1 only).

The reason could be that the film clips might have been simply too short. For reasons of comparability with the pictorial stimuli and to exclude confounding effects of stimulus duration, we produced film clips with a length of 6 s each, which is the typical presentation time for IAPS images, which is much shorter than clips used in other film studies (e.g., Gross and Levenson, 1995: average length 151 s; Hewig et al., 2005: average length 113 s; Bartolini, 2011: average length 206 s). However, the induction of emotions by film clips is a process even more complex than elicitation of emotions by pictures, since it not only comprises visual, but multimodal afferent information processing in real-time with both explicit and implicit information. The film‘s narration is supported not only by perceptual elements (i.e., lighting, camera-angle or the use of colors) but also by its dramaturgic structure. The entire effect of a film evolves during the playing time and peaks at its ending. Though a film might cause approximate affects within a single sequence or scene and even within one shot, a strict limitation of a clip to 6 s, regardless of its dramaturgic form, might narrow down its emotional power. The most effective results, in terms of emotional response, were achieved by “timeless” sequences without fundamental dramaturgic function (e.g., *Amélie, Along came Polly*) or sub-sequences that hadn't been noticeably edited (e.g., *Mr. Bean, Bruce Almighty*). In negative clips, the strongest results were achieved when actions were shown in total (e.g., *American History X, Texas Chain Saw Massacre*). Whereas static images show the results of a previous action, the film sequences usually rely on the action part as being the emotion elicitor.

A further reason might be that the photographs used show real humans suffering whereas Hollywood films tell fictitious stories, which allows for a greater emotional distance. However, if this was the case, then why were there no differences in producing positive emotions? Either the positive film clips were also too short (which means that longer positive clips would have the potential to elicit much stronger emotions than pictures) or there are other unknown valence-specific reasons. Recent findings suggest that positively and negatively valenced emotion ratings are influenced conversely by the length of film clips (Bartolini, 2011).

Another possibility is the potential effect of novelty of the stimuli on the strength of emotional modulation. Whereas the film clips in larger parts were known to the participants (clips from “Hollywood movies”), IAPS and the additional pictures were typically unknown prior to the presentation. It is well known that novel or deviant stimuli in general elicit larger brain responses, like, e.g., the P300 and late sensory components of pain related potentials (Sutton et al., 1965; Legrain et al., 2002). Since these potentials reflect responses to phasic sensory stimuli, other stimuli that contain emotionally relevant content may yield increased responses due to reinforcement mechanisms (Rolls, 1992). Since the modulatory effect was not generally less for the film condition but differentially changed (mostly equally or less effective than pictures, however more effective to elicit arousal in clips with positive content), novelty alone is unlikely to be the main reason for the findings.

### The 3-picture condition

In assessing the 3-picture variant, results showed no advantage over the classic 1-picture condition regarding the induction of positive mood, and, in terms of arousal induction, there may be even a slight disadvantage in comparison to the film condition (set 1 only). Nevertheless, to induce a state of negative mood, the 3-picture condition turned out to be significantly more effective than films and, at least in women, the classic 1-picture version. This is supported by the arousal ratings following the presentation of the 3pic condition, which in both sets and for both sexes were consistently the highest. In relation to the finding that pictures were more effective than films, this could be due to the possibility that multiple images add up to an even stronger effect in the sense of “more images—more impact.”

### Limitations of the study

Positive stimuli did not induce a significant positive effect on valence ratings, regardless of whether picture or film, whereas negative stimuli resulted in a highly significant effect toward the negative end of the scale compared to baseline ratings (mean: 6.2).

This means that participants were already in a mildly positive mood before the experiment began (which may be due to the perspective of being able to participate in a diverting experiment instead of attending their usual lecture) which could not, on average, be increased by the experimental manipulation to a significant amount. One reason could be that it may be easier to experimentally produce negative affective states than positive ones. This assumption is supported by a meta-analysis (Westermann et al., 1996) in which the authors state that at least half of the included emotion induction methods are significantly more effective for negative than for positive mood states. Another reason, which is not independent from the first, could be that the positive emotion induced by positive film clips and pictures was just in line with and hence completely “absorbed” by the prevailing (positive) mood, in other words a ceiling effect, while the negative stimuli benefitted from being in contrast to the baseline state of mood. To exclude the possibility that the results were distorted due to the elevated baseline mood, further analyses were performed including control for the pre-experimental state of emotion. However, the results were left unaffected.

A second potential limitation of this study concerns the exclusivity of explicit measures used. Pictures with affective contents do not only yield responses in form of valence and arousal ratings but can also elicit autonomic and cortical reactions as well as reflex behaviors, with the startle reflex perhaps as best candidate to reflect implicit emotional processing (Bradley et al., 2001a; Walla et al., 2011). As this study used explicit measures only, none of these responses were recorded. Although effects of the affective stimuli could be shown by the use of explicit measures and were discussed in detail, a study further investigating this topic using implicit measures in addition to explicit ratings could be of additional value.

Furthermore, the film clips used in this study have not been previously validated, and still captures from the validated movie clips might serve as a potential additional control.

## Conclusions

Several conclusions can be drawn from the data obtained in this study. First of all and most importantly, short film clips seem to be an effective method for eliciting emotions. Secondly, short film clips do not appear to be better than pictures at evoking emotion and arousal states, but further research needs to be done to see whether this result can be replicated. The question of whether this is due to the length of the film clips or to inherent stimulus qualities, such as the artificiality of films, and if there are valence-specific effects, also needs to be addressed. If the effects found for the explicit consciousness based measures are also true for implicit measures (e.g., autonomic responses) needs to be investigated.

## Author contributions

All authors listed, have made substantial, direct and intellectual contribution to the work, and approved it for publication.

## Funding

This study was supported by Deutsche Forschungsgemeinschaft DFG KFO 256-IP6 and SFB 1158.

### Conflict of interest statement

The authors declare that the research was conducted in the absence of any commercial or financial relationships that could be construed as a potential conflict of interest.

## Footnotes

^1^A film classification in terms of concrete emotions was conducted to assure a certain variance within each valence and for reasons of further research. Table 1 comprises this classification. However, for this study it is of no concern.

^2^IAPS numbers 2095, 2205, 2703, 2730, 3000, 3010, 3015, 3051, 3060, 3063, 3071, 3130, 3170, 3180, 3181, 3261, 3266, 3350, 3530, 3550.1, 4606, 4607, 4608, 4611, 4626, 4641, 4651, 4653, 5626, 5629, 5760, 5833, 6212, 6260, 6510, 6540, 6560, 6830, 8186, 9040, 9050, 9250, 9252, 9253, 9410, 9433, 9560, 9570, 9571, 9622, 9800, 9810. The additional pictures are provided by the authors by request.

^3^IAPS numbers 1463, 1811, 2070, 2080, 2095, 2170, 2205, 2340, 2341, 2360, 2550, 2655, 2703, 2730, 3000, 3005.1, 3010, 3015, 3051, 3060, 3063, 3071, 3101, 3130, 3150, 3170, 3180, 3181, 3230, 3261, 3266, 3350, 3400, 3500, 3530, 3550.1, 4606, 4607, 4608, 4611, 4626, 4641, 4651, 4653, 4656, 5621, 5626, 5629, 5760, 5833, 6212, 6260, 6300, 6360, 6510, 6540, 6560, 6821, 6830, 7380, 8080, 8170, 8186, 8190, 8210, 8370, 8400, 9040, 9050, 9220, 9250, 9252, 9253, 9300, 9405, 9410, 9433, 9560, 9570, 9571, 9582, 9592, 9594, 9600, 9622, 9635.1, 9800, 9810, 9910, 9921. The additional pictures are provided by the authors by request.
