# Supplementary material for: Emotion Elicitation: A Comparison of Pictures and Films
Source: Front Psychol. 2016 Feb 17;7:180. doi: 10.3389/fpsyg.2016.00180 (PMC4756121; doi:10.3389/fpsyg.2016.00180)
Supplement: Supplementary file 1 [file Table1.pdf]

Supplemenary Table 1: Results of One- and Two-way Anovas with repeated measures and Follow up Comparisons for different Set, Emotion Condition and Gender

		Valence					Arousal Set 1					Arousal Set 2				
		df	F	p	η^2	d	df	F	p	η^2	d	df	F	p	η^2	d
**Condition**	**One-way Anova reapeted measures ##**															
	within subject effects	2.45	480.54	0.00	0.79											
	**Follow-up comparison condition #**															
	Baseline vs. 1pic negative emotion			0.00		2.92										
	Baseline vs 3pic negative emotion			0.00		2.95										
	Baseline vs. Film negative emotion			0.00		2.20										
	Baseline vs. 1pic positive emotion			1.00		0.03										
	Baseline vs. 3pic positive emotion			1.00		0.12										
	Baseline vs. Film positive emotion			1.00		0.13										
**Set**	**Two-way Anova repeated measures ##**															
	Set 1 vs. Set 2, between effects	1.00	1.68	0.20	0.01											
	Condition * Set, within subject effects	1.00	4.76	0.29	0.03											
	Condition, within subject effects	1.00	6.66	0.11	0.46											
**Negative**	**Follow up comparisons Set (posthoc LSD)**															
**Emotion**	1pic vs. 3pic (male and female)	137.00	1.43	0.23												
	1pic vs. Film (male and female)	137.00	0.55	0.46												
	3pic vs. Film (male and female)	137.00	0.30	0.59												
**Positive**	**One-way repeated measures Anova ##**															
**Emotion**	condition	1.82	2.68	0.76	0.02											
	**Follow up comparison #**															
	1pic vs. 3pic (male and female)			0.051		0.13										
	1pic vs. Film (male and female)			0.16		0.04										
	3pic vs. Film (male and female)			1.00		0.00										
**Negative**	**Two-way repeated measures Anova ##**															
**Emotion**	condition	1.63	196.29	0.00	0.59		1.00	58.00	0.00	0.46		1.40	5.20	0.02	0.07	
	gender	1.00	12.01	1.00	0.08		1.60	9.40	0.003	0.12		1.00	8.90	0.004	0.12	
	condition & gender	1.63	4.27	0.02	0.03		1.60	5.30	0.01	0.07						
	**Follow-up comparison gender #**															
	1pic vs. 3pic (male and female)			0.30		0.11			0.00		0.30			0.00		0.17
	1pic vs. Film (male and female)			0.00		0.89			0.00		0.52					
	3pic vs. Film (male and female)			0.00		0.97			0.00		0.82			0.02		0.33
	1pic vs. 3pic (female only)			0.00		0.18										
	film vs 1pic & 3pic (male and female)			0.00		0.83										
	**Follow-up comparison for condition & gender #**															
	1pic vs. 3pic (male)								0.01		0.19					
	1pic vs. Film (male)								0.01		0.33					
	3pic vs. Film (male)								0.001		0.52					
	1pic vs. 3pic (female)								0.00		0.45					
	1pic vs. Film (female)								0.00		0.73					
	3pic vs. Film (female)								0.00		1.22					
**Positive**	**One-way repeated measures Anova ##**															
**Emotion**	condition						1.60	5.90	0.01	0.08		1.80	21.80	0.00	0.25	
	gender	1.00	12.12	0.001	0.08							1.00	3.90	0.52	0.06	
	**Follow-up comparison for condition**															
	1pic vs. 3pic (male and female)													0.00	0.41	
	1pic vs. Film (male and female)													0.00	0.53	
	3pic vs. Film (male and female)								0.02	0.22						

# t-test pairwise, Bonferroni corrected; ## within subject effects; Greenhouse-Geisser-corrected values are reported; Condition: 1pic, 3pic and film, Sample Size n= 139: 83, d Cohen’s d effect size
